# Writing a Systematic Review for Publication in a Health-Related Degree Program

**DOI:** 10.2196/15490

**Published:** 2019-10-14

**Authors:** Clemens Scott Kruse

**Affiliations:** 1 School of Health Administration Texas State University San Marcos, TX United States

**Keywords:** systematic review, health information management

## Abstract

**Background:**

The protocol in this manuscript was designed to help graduate students publish. It is the result of a challenge from our provost in 2013. I developed this protocol over the last 6 years and have exercised the protocol for the last 5 years. The current version of the protocol has remained mostly static for the last 2 years—only small changes have been made to the process.

**Objective:**

The objective of this protocol is to enable students to learn a valuable skill of conducting a systematic review and to write the review in a way that can be published. I have designed the protocol to fit into the schedule of a traditional semester, but also used it in compressed semesters.

**Methods:**

An image map was created in HTML 5.0 and imported into a learning management system. It augments traditional instruction by providing references to published articles, examples, and previously recorded instructional videos. Students use the image map outside the classroom after traditional instruction. The image map helps students create manuscripts that follow established practice and are reported in accordance with the Preferred Reporting Items for Systematic Reviews and Meta-Analyses (PRISMA), and whose authorship follows guidelines by the International Committee of Medical Journal Editors.

**Results:**

Since its inception, this protocol has helped 77 students publish 27 systematic reviews in nine journals worldwide. Some manuscripts take multiple years to progress through multiple review processes at multiple journals submitted in sequence. Two other professors in the School of Health Administration have used this protocol in their classes.

**Conclusions:**

So far, this method has helped 51% of graduate students who used it in my graduate courses publish articles (with more manuscripts under consideration whose numbers have remained uncounted in this sum). I wish success to others who might use this protocol.

## Introduction

### Background

Responding to a challenge by our provost to help our graduate students publish, I created this protocol to conduct and write high-quality systematic reviews that would not only serve as springboards to larger research, but also be publishable on their own. With this protocol, I created a process map to define each step. From the process map, I created an image map in HTML 5 (Multimedia Appendix 1) to be hosted online. The image map is hosted by Texas State University’s learning management system (LMS) and integrated into courses. It contains helper videos, articles, and examples to help guide the students outside the classroom. My process has been integrated into multiple courses at both the graduate and undergraduate levels. Between semesters, I enter into partnership with those graduate students whose work is of high quality and also research viable topics for the following term. With this protocol, I have helped 77/151 (51%) graduate students publish 27 systematic reviews [1-27] (two in press) in nine high-quality journals worldwide over the last 5 years: Journal of Medical Internet Research [2,4,8-11,14,15,17,18,26], Journal of Medical Systems [19,20,23,24], Journal of Telemedicine and Telecare [3,22], Technology and Healthcare, [5,6], BMJ Open [16,21], JRSM Open [12], Applied Clinical Informatics [25], Journal of Rehabilitation Medicine [27], and American Journal of Tropical Medicine and Hygiene [1].

### Why Write a Systematic Review?

**Figure 1:**
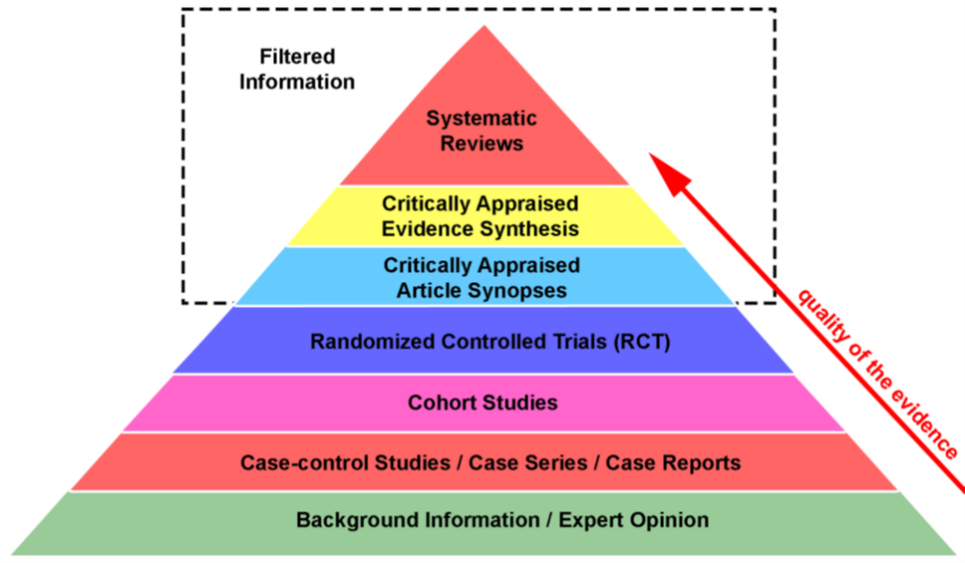
Levels of evidence analyzed in a systematic review.

Unlike a literature review for a report or early research, a systematic review looks carefully at a body of literature based on a highly specific question. It is “a comprehensive, transparent, and systematic literature review method for preparing, maintaining, and disseminating high-quality evidence” [28]. Systematic reviews can be used in any industry, but they are most often seen in the medical field, and they often comprise effectiveness questions of specific interventions [28]. A systematic review can be qualitative or quantitative in nature, and it can extend into a meta-analysis with some additional data extraction during the analysis phase. A systematic review uses “explicit, systematic methods that are selected to minimize bias, thus providing reliable findings from which conclusions can be drawn and decisions made” [29]. It reports its findings using an industry-accepted reporting mechanism such as PRISMA (Preferred Reporting Items for Systematic Reviews and Meta-Analyses) [29,30]. In addition, it examines a range of literature and filters as well as analyzes and summarizes the data into a condensed set of results and conclusions (Figure 1) [31].

## Methods

### Integrating a Systematic Review Into an Academic Course

Much of the timing and order of steps in this process revolve around a semester course in the Master of Health Administration program at Texas State University [32]. I integrate my process into the course that aligns with my primary area of research, The Management of Health Information Systems. I use the systematic review as the major deliverable for that course. I start the process on the first day of class because the process is already being compressed from a 12-month timeline recommended by the National Institute of Health [29]. A traditional semester compresses the process into four months, and a compressed semester fits it into only 5 weeks: This is a brutal schedule, but possible with careful guidance. Students must manage readings from the primary text for the course and those inherent to their review. Every class comprises 50%-60% text material and the remainder systematic review. During the systematic review portion of the course, the author provides about 15-20 minutes of instruction for the next step in the process. This instruction is reinforced on the image map with similar instructions previously recorded (Multimedia Appendix 1).

Texas State University has many resources available that the author requires for the systematic review. The LMS enables collaboration and document sharing. The library provides access to a variety of health-related databases such as PubMed, CINAHL (Cumulative Index to Nursing and Allied Health Literature), and CENTRAL (Cochrane Central Register of Controlled Trials). Students and faculty have access to Journal Citation Reports (JCR) and Cabell’s database. They also have access to RefWorks, Zoom, and Office 365. Regardless of student preferences, I require the use of university resources. Some groups prefer to use a service like Google Docs, but from my experience, students change access to these services between semesters, which hinders further refinement of the work later. The LMS serves as the document repository in case the author chooses to rewrite, expand, or build upon the review later as a coauthor or to invite other subject-matter experts to contribute. It is also ensures the group is maintaining proper workload and not procrastinating. I can also determine from the products uploaded if there are issues of communication difficulties or negative group dynamics and I can provide additional guidance to the group or to the class when it seems like the research is headed in the wrong direction. I also rewrite sections when the group submits them in a timely manner without penalty of grade, to incentivize timely submissions.

### Form Groups, Choose a Topic, Read on That Topic, Develop a Research Question

After introduction of the syllabus for the course, the author unveils and orients the students to the process map (Multimedia Appendix 1). Students self-form into groups of 3 or 4. Ideally, the group needs to have at least three people to enable a tie-breaker opinion [29]. Groups larger than four people tend to invite social loafers, which complicates group dynamics. I do not join immediately as a coresearcher because when I do, I tend to take over the project: This action does not enable the students to learn. I teach, mentor, and guide the students through the process throughout the semester. I help them overcome barriers; guide them through tough spots; and at times, mediate between group dynamics. I also use authorship guidelines from the International Committee of Medical Journal Editors (ICMJE) guidelines [33]:

- Substantial contributions to the conception or design of the work or the acquisition, analysis, or interpretation of data for the work;
- Drafting the work or revising it critically for important intellectual content;
- Final approval of the version to be published; and
- Agreement to be accountable for all aspects of the work in ensuring that questions related to the accuracy or integrity of any part of the work are appropriately investigated and resolved.

After the students self-form into groups, they sit in their groups for the remainder of the term. They also determine the roles for their team members: leader/project manager (to manage workload, set milestones and deadlines, and ensure regular group meetings using Zoom if necessary); editor (to work with RefWorks and write the review in one voice); literature matrix manager (to create and maintain the literature matrix in Excel); and for those groups with four members, a graphic artist (to create figures, tables, and other graphics in proper formats). I instruct the project manager to ensure documents integral to the review are uploaded to the LMS weekly. I also provide a set of deliverables and due dates to help them manage their time effectively. For instance, the literature matrix is an Excel document with several iterative tabs: Google Scholar search, abstract screening with Kappa, article analysis, themes, additional analysis, and charts.

Students conduct a basic search on their topic in Google Scholar. Google Scholar is not a research database, and its filter mechanisms are rudimentary. However, it serves as a good starting point for general research because it combines published research with grey literature. Students sort the results by date and every member of the group reads the top 10 articles; by the following class, they answer the following questions: What is my topic? What research has already been conducted on it? What research has not been conducted? The traditional-semester course meets once per week; therefore, the goal by the following week is for all members of the team to understand the topic. They identify 2-3 possible areas that have not been addressed through published research and could serve as their own research question. These 10 articles form the Introduction section of the systematic review, which includes the background, rationale, and objective(s) of the review, per the PRISMA checklist [30]. The literature matrix manager starts with a blank workbook and names several tabs that represent the major steps of the process. The Google Scholar tab of the literature matrix contains the following fields: date of publication, authors, study title, journal, impact factor, study design, key terms, technology intervention, results, observations from each group member, and a recommendation from each group member of whether to keep or reject each article in the analysis. These articles become the first 10 references.

### Journal Selection, Journal Formats, and Established Principles of Scientific Writing

I highly consult the JCR as a source of quality metrics for journals and ask students to enter this information into the literature matrix. Based on the entries in the literature matrix, students observe the journals already publishing on their topic, which are the journals most likely to publish on their topic again. I ask project managers to write to the editors of the top three journals publishing on their topic. An example of verbiage for this email is provided on the image map. Journal editors may send only a form response that they cannot opine on a topic without sending the article for peer review: This is not a “no.”

In the second-class meeting, students provide an article critique of an article already published from the course (links to all articles published in the course are included in a folder in the LMS and link through the university library to meet copyright requirements). The critique is focused on how closely the published article followed the PRISMA checklist. The purpose of this deliverable is to alleviate fears about this onerous project and to show the level of formatting that the journal’s typesetting process provides. The process map itself is intimidating (a repeated comment on the course critiques). The longer students procrastinate starting the systematic review, the lower the quality of the review and the lower the likelihood that it will be of sufficient quality for publication. The article critique shows the students that the process itself is systematic in its approach, but it is not insurmountable, and complex formatting is not required at the authors’ level. The critique illustrates that journals dictate the preferred writing style. The published articles do not completely adhere to every step of PRISMA, but they include most of them. The peer-review process often strips out entire subsections of the PRISMA checklist. Many subsections of PRISMA are combined to enable better flow of the manuscript. At the end of the critique, I ask students to follow PRISMA as closely as possible. If any portion of PRISMA does not apply to the reviews, they will at least address each step and state why it does not apply. By the end of the first week, students have created a preliminary literature matrix that documents details about and observations from the first 10 articles. The literature matrix lists and rank orders the top three journals most likely to publish their review. Students transcribe this information in a PowerPoint presentation titled “Author’s Guidelines” (Textbox 1). As depicted by the figure, the first line is a link to the journal’s page that provides all details of the guidelines. At the end of the semester, the author grades the format of what students write based on the Author’s Guidelines from the preferred journal.

As depicted by the image map in Multimedia Appendix 1, 12 articles published by the Journal of Clinical Epidemiology in 2013 are integrated into the process [35-46]. These articles walk readers through getting started, [35] title and summary [36], introduction [37], methods [38], results [39], discussion [40], references [41], authorship [42], selecting a journal [43], submitting the article [44], responding to reviewers [45], and tables and illustrations [46]. They are excellent articles that I teach from regularly. Many portions of my image map in Multimedia Appendix 1 were inspired by these articles. Due to the compressed schedule already dictated by an academic semester, students do not submit the article themselves or respond to reviewers as part of the assignment. If I enter into a coauthorship agreement with students, I serve as the corresponding author and handle all rewrites. I teach the students about these important steps in class, and after the course has ended, I keep them all informed of the article’s progress through the publication process, per ICMJE.

**Author's guidelines: a deliverable for early class period.**

**Choice 1: Applied Clinical Informatics**

- Author’s guidelines [34]
- Writing style: Vancouver
- Length limit: 2500-3000 words, not exceeding 5000 words
- Impact factor: 1.306 (2018)
- Listed in Journal Citation Reports or Cabell’s database: yes
- Acceptance rate: <50%
- Time to publish: unknown
- Open access fee for publication: US $2400

### Introduction Section and the Importance of a Specific Objective Statement

**Figure 2:**
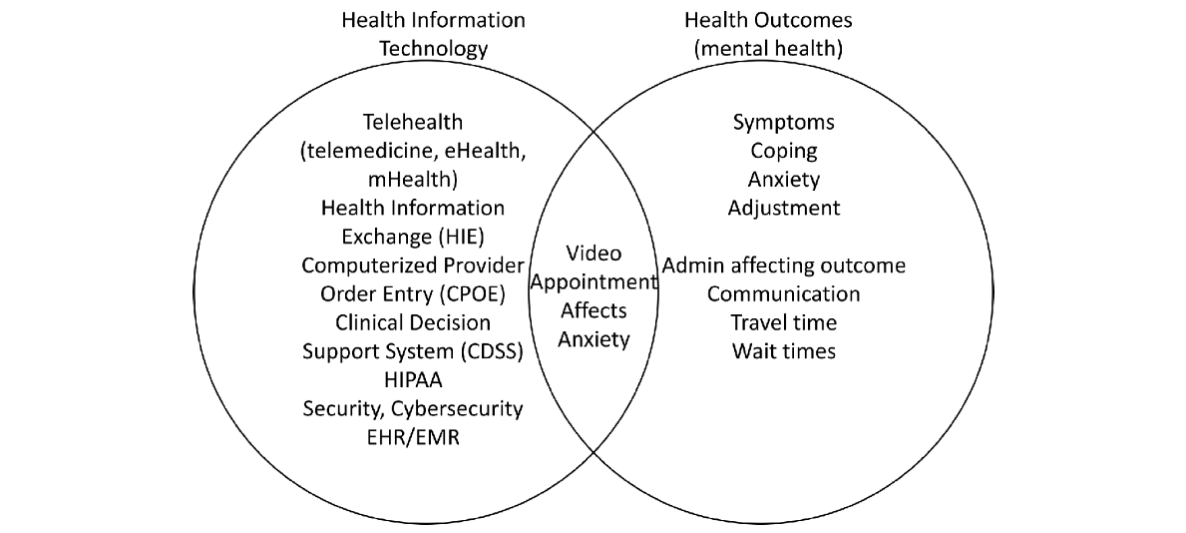
Venn diagram showing the intersection of major concepts where research is to be conducted.

The introduction section comprises a definition of major concepts and how they are related: This can be visualized in a Venn diagram (Figure 2). The first big concept is one application within health information technology, such as telemedicine or health information exchange (because that is the purpose of the course in which this deliverable is integrated). These definitions can come from the World Health Organization. The second big concept is one aspect of effectiveness and health outcomes such as reduced anxiety, improved patient-to-provider communication, increased adherence to treatment, fewer barriers to keeping an appointment, etc.

These definitions come from the professional organizations that care for the specific condition under study such as mental health, cardiovascular health, or prenatal care. The introduction defines these concepts and where they intersect. The introduction should list the research that has been conducted on this intersection so far, and it should culminate in what has not been studied (or needs to be studied further) [37]. This should be logically followed by the objective statement.

The objective statement is a specific point in the intersection of the important concepts. It clearly defines what the researchers intend to investigate. The objective statement should include all aspects of PICO (population, intervention, comparison, and outcomes) that are included on the second tab of the literature matrix as part of the analysis [29]. The importance of a clearly defined objective statement cannot be understated. This objective statement serves as a litmus test for group members to determine whether an article appeals to their purpose. Many articles are not properly indexed, and therefore, many are not germane to the research that students are trying to conduct. When students struggle to agree on articles to include in their analysis, I continually refer to the objective statement that they have written and agreed upon in the introduction section. I also help groups rewrite this section prior to moving onto the database searches.

### The Database Search, Abstract Screening, and Cohen Kappa

Referring to the literature matrix, students should have collected the key terms from the initial 10 articles from the introduction. These terms and Boolean operators serve as the starting point for what will be used to search the research databases. Beginning with these search terms, students refer to PubMed’s Medical Subject Headings (MeSH), because it lays out the hierarchy of index terms under which it classifies articles. When a student types in “health information technology,” for instance, some useful information is portrayed. This exact term is not indexed in PubMed, and therefore, a search that includes this key term would not be productive. However, changing the term to “health information management” yields richer results (Textbox 2). Under “entry terms,” the MeSH hierarchy shows that several similar terms would yield the same result, such as health information management, and that there are several parent terms to the one entered. There is one additional term listed below health information management.

A basic understanding of how MeSH indexes articles makes the search string more effective at identifying articles appropriate for analysis. If a student clicks on the last term, “health information exchange” additional information is portrayed. If a student group wanted to analyze the effect of health information exchange on readmission rates to emergency rooms, they would know that similar terms such as “medical information exchange” would already be included, which simplifies an exhaustive search string. I encourage students to experiment with several combinations of their search terms to familiarize themselves with MeSH and ensure their search string is exhaustive in PubMed.

Students filter their results below 100 results using filters such as the last 10 years; omit other reviews; and if necessary, only include those in full text. The same search string is used in at least one other database. I recommend CINAHL and CENTRAL, and in those databases, remove MEDLINE, which takes care of most of the duplication with PubMed. When both CINAHL and CENTRAL are used, there is a great deal of overlap, which complicates the removal of duplicates. This is particularly problematic when there are several hundred articles to organize. Students download results from all databases to a csv file, rearrange columns so that they match, and then combine all results into one worksheet in the literature matrix to enable analysis. Students remove duplicates and screen abstracts for appropriateness to their objective statement. Students also download abstracts to a simple text file, matching the numbers to the literature matrix. This makes it easy for group members to screen abstracts. The project manager divides workload on the literature matrix with Xs next to the article entry, so that two group members review each abstract and opine on its appropriateness. This technique is supported by the Assessment Methodology Quality of Multiple Systematic Reviews (AMSTAR) [47]. An example of the workload allocation is provided in the image map (Table 1). The author asks the literature matrix manager to add a column for him, so that he can randomly screen articles on his own as part of the process. This enables him to render assistance when the group has difficulty reaching agreement.

**Medical Subject Headings term search result.**

**Entry terms:**

- Health Information Managements
- Information Management, Health
- Information Managements, Health
- Management, Health Information
- Managements, Health Information All MeSH Categories
- Information Science Category
- Information Science Information Management Health Information Management Health Information Exchange

**Table 1:** Division of workload. “X” indicates which article is being reviewed.

	Student Reviewer 1	Student Reviewer 2	Student Reviewer 3	Author	Final decision
Article 1	X	X			
Article 2	X	X		X	
Article 3	X	X			
Article 4		X	X	X	
Article 5		X	X		
Article 6		X	X	X	
Article 7	X		X		
Article 8	X		X	X	
Article 9	X		X		

**Figure 3:**
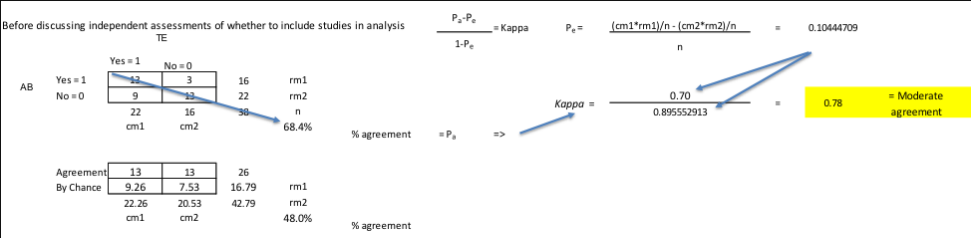
Calculation of kappa statistic using a series of Chi-square tables.

The editor of the group should write a statement of qualification from the objective by which students can screen their abstracts; for example, articles qualify for analysis in the systematic review if they analyzed cardiovascular health using one or more interventions of telehealth or document health medical outcomes and were published in the last 10 years. Over the next week, between class meetings, students in the group will screen abstracts to determine if they are germane to their objective and replace the “X” of workload assignment with a “1” or “0,” indicating keep or reject. By the end of the screening process, students typically end up with a group for analysis between 30-60 assignments. This is a good point to register the review with a service like PROSPERO (International Prospective Register of Systematic Reviews), because it is far enough along to have a firm objective statement, but it is prior to analysis being performed. Prior to the next class session, the project manager holds the first “consensus meeting” in which the group discusses disagreements and makes a final determination on the group of articles for analysis. Discord is alleviated by a third member reading the abstract. Based on the group’s recommendations, the PRISMA flow diagram can be started. Based on the 1s and 0s in the literature matrix, I calculate the Cohen kappa statistic using a series of Chi-square tables (Figure 3 and Multimedia Appendix 2) [48,49].

The literature matrix manager creates a third tab in the workbook with the final group for analysis and pastes those articles chosen for analysis from the previous tab. The project manager allocates workload, ensuring each article is analyzed by two reviewers in the same manner as the screening of abstracts. I ask the literature matrix manager to include a column for him as well to provide additional analysis. The literature matrix serves as the data extraction tool. The fields in the worksheet are database, date of publication, journal, authors, title, population, technological intervention, study type, comparison/control, outcomes, sample size, bias within study, effect size, country of origin, and anything else of relevance to the objective. Reviewers should also scan the references to identify articles missed by our search. External to workload assignments, I randomly choose articles in the list and provide my own observations. This variety of fields enables a thorough analysis of the articles in the review and the identification of common themes throughout the articles. It provides enough data to present visually through charts and enables a meta-analysis later.

This is the longest phase in the process (approximately 3 weeks), and it includes at least two additional consensus meetings. The second consensus meeting should enable group members to discuss what they have found so far, identify any additional articles from references that the database search missed, and discuss potential themes (common threads through articles). This step occurs only 2-3 weeks after the start of the course, so realistically, students are still figuring out their topic and how to conduct a systematic review. I realize this reality and provide regular coaching and class exercises to keep the students on task. Students will read all articles assigned to them while collecting data. I suggest the students read each article a second time after the first consensus meeting to identify additional themes that occur between articles. Themes will not surface until several articles have been read and analyzed. It takes multiple reads to thoroughly digest the group of articles. The third consensus meeting should identify the final set of themes and group the observations into these themes; for example, cost savings and additional expenditures are both “cost” and improved mobility and increased range of motion are both “improved health outcomes.” Grouping into themes simplifies data summary and additional analysis.

## Results

The literature matrix contains all the data extracted from the articles, and as such, it serves as the source for tables, charts, etc. Typically, the first table included in the review provides the observations and themes identified in the articles. Another table contains PICO and observed bias (I recommend this serve as an appendix for journals that do not offer tables in landscape view). An affinity matrix can be created to identify themes throughout the group for analysis, countries of origin, outcomes reported, and anything else of significance. As a rule, I recommend the results section be terse but informative. A table should be included that lists the articles organized by date, newest to oldest, with a brief sentence of the analysis performed pertinent to the objective statement. The sentence that introduces the table should list all the articles as references. This is important because many journals must have a requirement to list the article outside of a table, and by listing them in order by date, this order is now set for discussion. As previously mentioned, a more extensive table organized by PICO should be included as an appendix. Discussion and interpretation of the results should be reserved for the Discussion section.

## Discussion

### Overview

This protocol was designed to help graduate students publish a systematic review in a high-quality, peer-reviewed journal. How the protocol addresses each section has been detailed and examples have been provided. This protocol has been proven with a history of successfully published articles over time, but end products are only as good as the level of effort invested into the analysis of articles.

The PRISMA checklist identifies several subsections for the Discussion section: Summary of evidence (which is where analysis of results takes place), limitations (and bias), and conclusions (with interpretation of results) [30]. The Discussion section is also the place for suggesting future research and comparison to previous research. The final section in the PRISMA checklist is the funding paragraph. Each journal has specific wording for this paragraph that can be found in the Guidelines for Authors.

### Helping Graduate Students Publish

At the end of each term, I grade work based on process, format, and substance. I do not grade harshly because I do not expect students to be proficient in writing for publication at the master’s level. After entering grades for the work the students have done, I assess each article for its potential to be published. As in any program, not all groups take the assignment seriously. This seems to occur about 25% of the time. These groups follow the process and generally do what they were asked, but often, their analysis is shallow. Occasionally, a group omits a key term that radically changes the search results and group for analysis. Depending on the workload, I prioritize the articles from most to least potential and enter into a coauthorship agreement with the students. I spend my semester breaks duplicating the search results, analyzing all articles, and discerning meaning. I fine-tune graphics and ensure all formatting meets the requirements of journals. I then carefully comb through each section, strengthening the conclusions. I ensure the standard of the product meets those set by the editor. My level of effort to rewrite the product determines where I put my name on the authorship line. I send out the final version of the manuscript to the students prior to submitting to a journal. I obtain permission from the school Chair to fund the article using Open Access and then submit the article and track its progress. Some articles take years to matriculate through the publication process [11]. Others take only a couple months [26].

### Continual Improvement to the Process Map

I continue to make small modifications to the process where needed, but it has remained in its current state now for about 2 years. Due to its static nature, it was the right time to share what I have used. At the university in which I teach, I created an LMS project site with the image map and all related resources. This can be imported into other courses and appears as a navigation button. So far, only two other professors have used it in this manner, but it is available for others upon request (access must be granted to the site).

### Limitations

There are many limitations to conducting systematic reviews, but this protocol addresses and overcomes many of them. Selection bias is addressed through exhaustive searches with MeSH terms, using the same search string in multiple databases and by using multiple reviewers. This is supported by AMSTAR [47]. Publication bias is addressed by including grey literature in the search.

### Conclusions

I have found success with this method, and peers have asked me to write this article as both an aide for students and other professors who want to use the image map in their teaching. So far, this method has helped 77/151 (51%) graduate students publish with iterations of this protocol. Two manuscripts are in press, one is under consideration on its second peer review, two manuscripts are under consideration on their initial peer review, and three are in progress. I wish success to others who might use this protocol.

## Appendix

Multimedia Appendix 1 HTML5 image map that provides instructions to augment teaching in classroom. URL to dynamic code; https://h5p.org/h5p/embed/28817.

Multimedia Appendix 2 Literature matrix Excel worksheet, second tab, and "abstract screening", from which a kappa statistic is calculated.

## Abbreviations

AMSTAR: Assessment of Methodological Quality of Multiple Systematic Reviews
CENTRAL: Cochrane Central Register of Controlled Trials
CINAHL: Cumulative Index to Nursing and Allied Health Literature
ICMJE: International Committee of Medical Journal Editors
JCR: Journal Citation Reports
LMS: Learning Management System
MeSH: PubMed Medical Subject Headings
PICO: population, intervention, comparison, and outcomes
PRISMA: Preferred Reporting Items for Systematic Reviews and Meta-Analyses
PROSPERO: International Prospective Register of Systematic Reviews
