# Supplementary figures and images for: Writing a Systematic Review for Publication in a Health-Related Degree Program
Source: JMIR Res Protoc. 2019 Oct 14;8(10):e15490. doi: 10.2196/15490 (PMC6914304; doi:10.2196/15490)

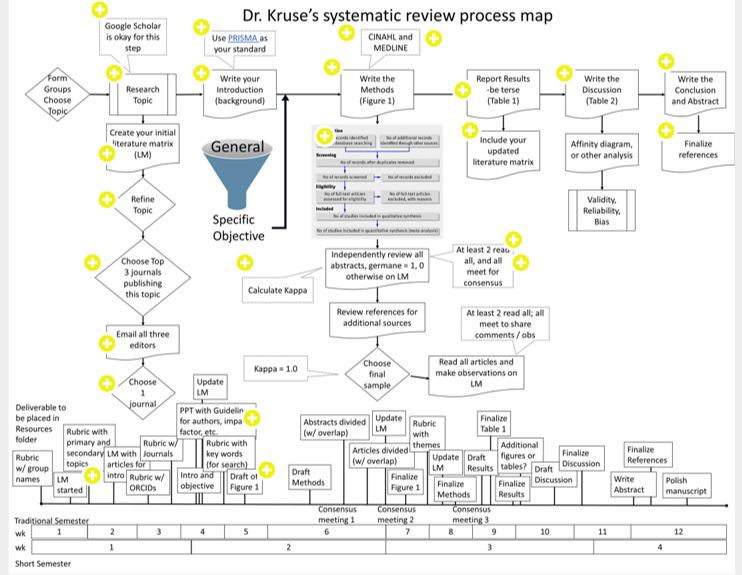

Supplement: Multimedia Appendix 1 [file resprot_v8i10e15490_app1.png]
